# SharePro: an accurate and efficient genetic colocalization method accounting for multiple causal signals

**DOI:** 10.1093/bioinformatics/btae295

**Published:** 2024-04-30

**Authors:** Wenmin Zhang, Tianyuan Lu, Robert Sladek, Yue Li, Hamed Najafabadi, Josée Dupuis

**Affiliations:** Quantitative Life Sciences Program, McGill University, Montreal, Quebec H3A 1E3, Canada; Montreal Heart Institute, Université de Montréal, Montreal, Quebec H1T 1C8, Canada; Department of Statistical Sciences, University of Toronto, Toronto, Ontario M5S 1A1, Canada; Quantitative Life Sciences Program, McGill University, Montreal, Quebec H3A 1E3, Canada; Department of Human Genetics, McGill University, Montreal, Quebec H3A 0C7, Canada; Dahdaleh Institute of Genomic Medicine, McGill University, Montreal, Quebec H3A 0G1, Canada; Quantitative Life Sciences Program, McGill University, Montreal, Quebec H3A 1E3, Canada; School of Computer Science, McGill University, Montreal, Quebec H3A 2A7, Canada; Quantitative Life Sciences Program, McGill University, Montreal, Quebec H3A 1E3, Canada; Department of Human Genetics, McGill University, Montreal, Quebec H3A 0C7, Canada; Dahdaleh Institute of Genomic Medicine, McGill University, Montreal, Quebec H3A 0G1, Canada; Quantitative Life Sciences Program, McGill University, Montreal, Quebec H3A 1E3, Canada; Department of Epidemiology, Biostatistics and Occupational Health, McGill University, McGill College, QC H3A 1Y7, Canada

## Abstract

**Motivation:**

Colocalization analysis is commonly used to assess whether two or more traits share the same genetic signals identified in genome-wide association studies (GWAS), and is important for prioritizing targets for functional follow-up of GWAS results. Existing colocalization methods can have suboptimal performance when there are multiple causal variants in one genomic locus.

**Results:**

We propose SharePro to extend the COLOC framework for colocalization analysis. SharePro integrates linkage disequilibrium (LD) modeling and colocalization assessment by grouping correlated variants into effect groups. With an efficient variational inference algorithm, posterior colocalization probabilities can be accurately estimated. In simulation studies, SharePro demonstrated increased power with a well-controlled false positive rate at a low computational cost. Compared to existing methods, SharePro provided stronger and more consistent colocalization evidence for known lipid-lowering drug target proteins and their corresponding lipid traits. Through an additional challenging case of the colocalization analysis of the circulating abundance of R-spondin 3 GWAS and estimated bone mineral density GWAS, we demonstrated the utility of SharePro in identifying biologically plausible colocalized signals.

**Availability and implementation:**

SharePro for colocalization analysis is written in Python and openly available at https://github.com/zhwm/SharePro_coloc.

## 1 Introduction

Colocalization analysis is a commonly used statistical procedure to assess whether two or more traits share the same genetic signals identified in genome-wide association studies (GWAS) (Giambartolomei *et al.* 2014, Hormozdiari *et al.* 2016, Wen *et al.* 2017, Zheng *et al.* 2020, Wallace 2020, 2021, Hukku *et al.* 2021). It is important for understanding the interplay between heritable traits (Pickrell *et al.* 2016, Sun *et al.* 2018), such as validating causal inference results based on Mendelian randomization analysis (Richardson *et al.* 2020, Zheng *et al.* 2020, Zuber *et al.* 2022) and identifying candidate genes for functional follow-up studies (Fortune *et al.* 2015, Hormozdiari *et al.* 2016, Lu *et al.* 2023, Yoshiji *et al.* 2023). Therefore, a powerful colocalization method with a well-controlled false positive rate is crucial for increasing the yield of complex trait genetics studies.

COLOC (Giambartolomei *et al.* 2014) is one of the most widely used methods for colocalization analysis. COLOC uses a Bayesian framework to estimate posterior probabilities of five different causal settings in a locus (H0: no causal signal; H1: one unique causal signal for trait 1; H2: one unique causal signal for trait 2; H3: different causal signals for trait 1 and trait 2; H4: one shared causal signal for trait 1 and trait 2.). Colocalization probability is defined as the posterior probability of H4 (Giambartolomei *et al.* 2014). A key assumption in COLOC is that only one causal variant exists for each trait in a genomic locus (Giambartolomei *et al.* 2014). In both simulation and substantive studies (Giambartolomei *et al.* 2014, Fortune *et al.* 2015), COLOC demonstrated high accuracy in identifying the shared causal signal when the one-causal-variant assumption was met. However, the performance of COLOC may be compromised when more than one causal signal exists in a genomic locus (Hormozdiari *et al.* 2016, Nilsson *et al.* 2021, Wallace 2021).

Building upon COLOC, several methods have been developed to address these challenges. For example, eCAVIAR allows for multiple causal signals (Hormozdiari *et al.* 2016) by adopting the CAVIAR (Hormozdiari *et al.* 2014) fine-mapping framework for colocalization. In eCAVIAR, colocalization is assessed at the variant level by examining the probabilities of variants being causal in both traits. Specifically, the posterior inclusion probabilities for variants are first calculated separately for each trait. Then, the variant-level colocalization probabilities are obtained as the product of the posterior inclusion probabilities. Assuming a consistent genome-wide enrichment of molecular quantitative trait loci (QTL) in GWAS across different loci, ENLOC/fastENLOC uses a multiple imputation strategy to estimate global enrichment parameters for specifying optimal prior colocalization probabilities to further improve the performance of COLOC and eCAVIAR (Wen *et al.* 2017, Hukku *et al.* 2021). Recently, COLOC + SuSiE (Wallace 2021) adopts a fine-mapping method SuSiE (Wang *et al.* 2020) for identifying multiple causal variants before performing pairwise colocalization, which could improve the performance of COLOC when multiple causal signals exist. Similarly, PWCoCo (Robinson *et al.* 2022) first performs conditional and joint analysis with GCTA-COJO (Yang *et al.* 2012), followed by colocalization analysis on each pair of the conditionally independent signals identified by GCTA-COJO using COLOC. These methods implement a two-step strategy. Namely, they first account for LD via fine-mapping or conditional analysis to identify candidate variants for colocalization analysis, separately for each trait. And then, under the one-causal-variant assumption, colocalization probabilities are assessed by examining whether each pair of candidate variants represents the same causal signal. However, with this strategy, the uncertainties in accounting for LD from the first step might affect the assessment of colocalization in the second step.

We propose SharePro (Shared sparse Projection for colocalization analysis) to integrate LD modeling and colocalization assessment and account for multiple causal variants in colocalization analysis. In SharePro, highly correlated variants are grouped into effect groups and colocalization probabilities are assessed by examining the causal status of each effect group in different traits. We evaluate the performance of SharePro in simulation studies in comparison to state-of-the-art colocalization methods. We utilize positive controls to benchmark the performance of existing colocalization methods in real data, including circulating proteins that are established lipid-lowering drug targets and their corresponding lipid traits. We further examine colocalization between the circulating abundance of R-spondin 3 (RSPO3) GWAS and a GWAS locus identified for estimated bone mineral density (eBMD) using heel quantitative ultrasound measurement to evaluate whether SharePro could better identify biologically plausible colocalized signals.

## 2 Materials and methods

### 2.1 SharePro method overview

**Figure 1.**
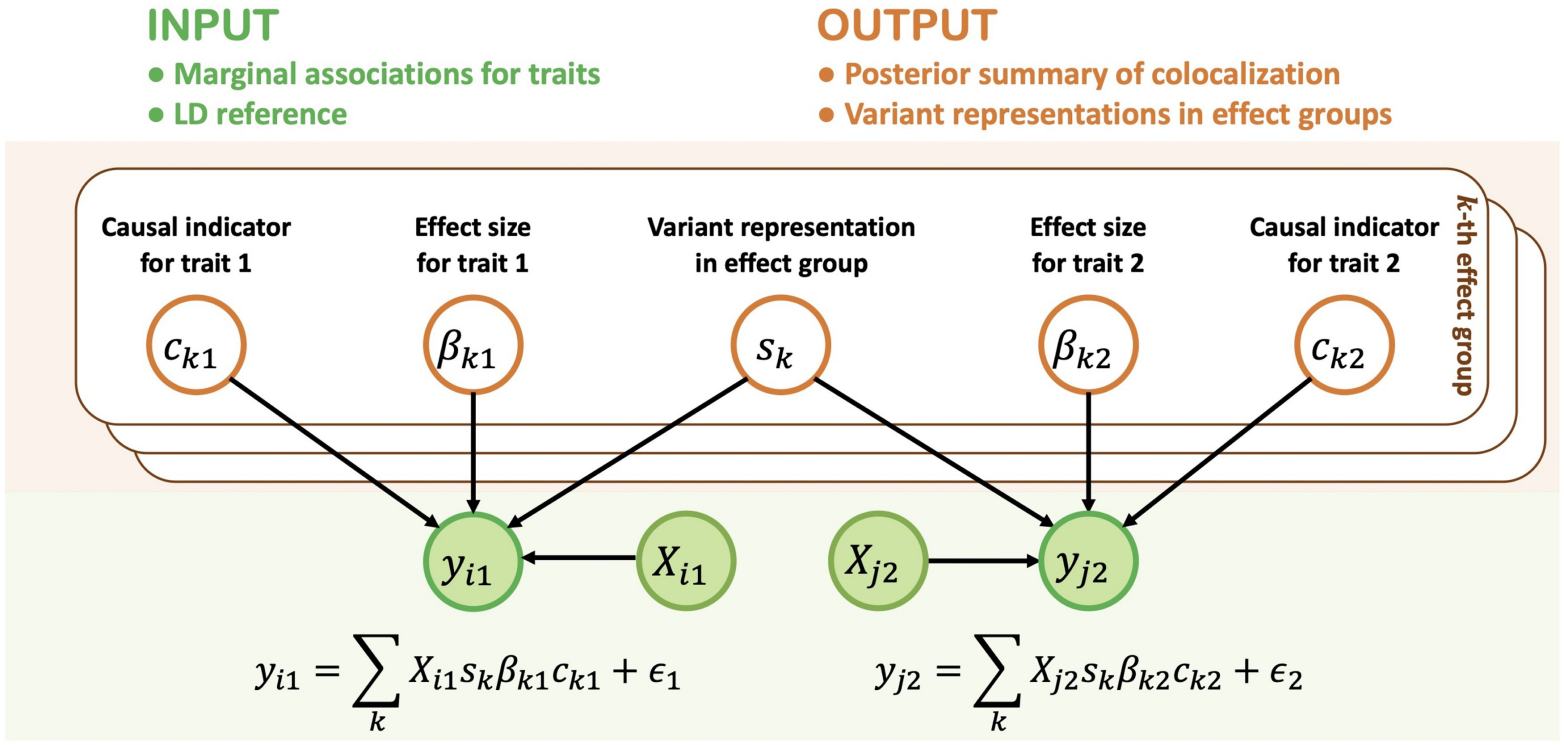
SharePro for genetic colocalization analysis. The data generative process in SharePro is depicted in the graphical model. Green shaded nodes represent observed variables: genotype $X_{i1}$, trait $y_{i1}$ for the *i*th individual in the first study, and genotype $X_{j2}$, trait $y_{j2}$ for the *j*th individual in the second study. The orange unshaded nodes represent latent variables characterizing effect groups. $s_{k}$ is a sparse indicator shared between traits, specifying variant representations for the *k*th effect group. $c_{k1}$ and $c_{k2}$ are causal indicators of whether the *k*th effect group is causal in trait $y_{1}$ and trait $y_{2}$ while $\beta_{k1}$ and $\beta_{k2}$ represent the corresponding effect sizes. As a result, colocalization probability for the *k*th effect group is the posterior probability of $c_{k1}=c_{k2}=1$. Here we assume individual-level data are available. Adaption to GWAS summary statistics is detailed in the Supplementary Note.

SharePro takes marginal associations (*z*-scores) from GWAS summary statistics and LD information calculated from a reference panel as inputs and infers posterior probabilities of colocalization (Fig. 1). Unlike existing methods, SharePro adopts an effect group-level approach for colocalization. Specifically, SharePro uses a sparse projection shared across traits to group correlated variants into effect groups. Through this shared projection, variant representations for effect groups are the same across traits so that colocalization probabilities can be directly calculated at the effect group level. With an efficient variational inference algorithm, both variant representations for effect groups and their causal statuses in each trait can be accurately inferred. Consequently, we can obtain colocalization probabilities from the posterior probabilities of effect groups being causal for all traits.

### 2.2 SharePro for colocalization analysis

In SharePro, we use effect group to represent a group of variants that are highly correlated with each other. The colocalization probability is assessed at the effect group level. In a locus with *G* variants, we assume there are *K* effect groups. Similar to our previous work on the sparse projection formulation of the SuSiE model (Wang *et al.* 2020, Zhang *et al.* 2023a), for the *k*th (k∈{1,…,K}) effect group, in SharePro, we use $s_{k}$, a sparse indicator shared by both traits to specify its variant representations (Fig. 1). This indicator follows a multinomial distribution:

$$s_{k}∼\mathrm{Multinomial}(1,1_{G\times 1}\times \frac{1}{G})$$

We use two additional sets of trait-specific variables to describe relationships between the $k^{th}$ effect group and each trait: causal indicators $c_{k1}$, $c_{k2}$ of whether the *k*th effect group is causal for $y_{1}$ and $y_{2}$ and $\beta_{k1}$ and $\beta_{k2}$ for their corresponding effect sizes (here we illustrate the model with two traits but it is also compatible with multiple traits):

$$\begin{array}{c} c_{k1},c_{k2}∼\mathrm{Bernoulli}(\sigma )\\ \beta_{k1}∼N(0,\tau_{\beta_{1}}^{-1})\\ \beta_{k2}∼N(0,\tau_{\beta_{2}}^{-1}) \end{array}$$

Denoting the genotype matrix as $X_{1}$ and $X_{2}$, for traits $y_{1}$ and $y_{2}$, we have:

$$\begin{array}{c} y_{1}∼N(X_{1}\sum_{k}s_{k}\beta_{k1}c_{k1},\tau_{y_{1}}^{-1}I)\\ y_{2}∼N(X_{2}\sum_{k}s_{k}\beta_{k2}c_{k2},\tau_{y_{2}}^{-1}I) \end{array}$$

$\tau_{\beta_{1}}$ and $\tau_{\beta_{2}}$ are hyperparameters for effect size distributions while $\tau_{y_{1}}$ and $\tau_{y_{2}}$ are hyperparameters for residual error distributions; σ is the important hyperparameter related to prior colocalization probability. We discuss choices of these hyperparameters in the Supplementary Note. The colocalization probability for the *k*th effect group is represented by the posterior probability of $p(c_{k1}=c_{k2}=1|y_{1},y_{2},X_{1},X_{2})$. We use an efficient variational inference algorithm (Titsias and Lazaro-Gredilla 2011, Blei *et al.* 2017, Zhang *et al.* 2023a) adapted for GWAS summary statistics for the posterior inference of the variant representations for effect group $p(s_{k}|y_{1},y_{2},X_{1},X_{2})$ and the posterior colocalization probability $p(c_{k1}=c_{k2}=1|y_{1},y_{2},X_{1},X_{2})$ as detailed in the Supplementary Note.

### 2.3 Simulation studies

We conducted simulation studies under different causal settings to evaluate the performance of colocalization methods. We randomly sampled five 1-Mb loci from the genome and extracted their genotypes for 25 000 and 1000 nonoverlapping UK Biobank European ancestry individuals (Bycroft *et al.* 2018) to simulate trait 1 and trait 2, respectively. For each locus, we calculated the LD matrix using PLINK (Purcell *et al.* 2007).

In each locus, we randomly sampled $K_{C}$ causal variants to be shared across traits and additionally $K_{S}$ causal variants to be trait-specific. For example, when $K_{C}=0$ and $K_{S}=1$, there was one causal variant for trait 1 and one different causal variant for trait 2; When $K_{C}=1$ and $K_{S}=0$, there was one causal variant shared by both traits. We set the per-variant heritability to be 0.01 in trait 1 and 0.05 in trait 2. With simulated traits, we performed GWAS using GCTA (Yang *et al.* 2011) to obtain summary statistics. We repeated this process 50 times for each of the five different loci, resulting in a total of 250 replications for each setting.

With LD information and simulated summary statistics, we performed colocalization analysis with five different methods (Table 1) using default prior colocalization probabilities. In SharePro, the maximum number of effect groups was set to 10. We obtained posterior colocalization probabilities from COLOC (Giambartolomei *et al.* 2014). Both COLOC+SuSiE (Wallace 2020) and PWCoCo (Robinson *et al.* 2022) generated multiple pairs of colocalization probabilities, with the maximum used as colocalization probabilities. For eCAVIAR, we also used the maximum variant-level colocalization probabilities as locus-level colocalization summary (Hormozdiari *et al.* 2016). Similarly in SharePro, maximum colocalization probabilities across all identified effect groups were used. Additionally, we performed prior sensitivity analysis for COLOC and SharePro to investigate the impact of prior colocalization probabilities in colocalization analysis.

**Table 1.** Summary of colocalization method features.

Method	Multiple causal variants	Signal identification	Posterior summary	Running time (second; SD)	Reference
COLOC	X	X	Locus-level	0.1 (0.1)	Giambartolomei *et al*. (2014)
COLOC+SuSiE	✓	Separate fine-mapping	Paired locus-level	14.0 (3.3)	Wallace (2021)
eCAVIAR	✓	Separate fine-mapping	Variant-level	227.7 (89.3)	Hormozdiari *et al*. (2016)
PWCoCo	✓	Conditional analysis	Paired locus-level	38.1 (20.5)	Zuber *et al*. (2022)
SharePro	✓	Joint fine-mapping	Effect group-level	4.3 (1.1)	This study

A colocalization probability >0.8 was considered strong evidence supporting colocalization, while a colocalization probability <0.2 was considered evidence against colocalization (Zheng *et al.* 2020). Moreover, we calculated the power of COLOC+SuSiE, eCAVIAR, and SharePro in identifying individual colocalized signals, defined as the proportion of simulated shared causal variants being included in any pair of variants (COLOC+SuSiE and eCAVIAR) or effect group (SharePro) with a colocalization probability >0.8.

### 2.4 Colocalization analysis of lipid-lowering drug target protein QTL and GWAS for lipid traits

We used several positive controls to evaluate the performance of different colocalization methods in identifying colocalized signals that have been biologically verified. We performed colocalization analyses based on GWAS for the circulating abundances of known lipid-lowering drug target proteins and GWAS for their corresponding lipid traits.

Lipid-lowering drugs and the corresponding genes encoding the pharmacological targets of these drugs were curated in Li *et al* (2023). The cis-protein QTL summary statistics were obtained from the Fenland study (Pietzner *et al.* 2021), where four of these lipid-lowering drug target proteins were available: APOB, PCSK9, APOC3, and ANGPTL3. Additionally, we obtained European ancestry-specific cis-protein QTL summary statistics for LPL from the UK Biobank (Sun *et al.* 2023), which was not measured in the Fenland study. European ancestry-specific GWAS summary statistics for serum low-density lipoprotein cholesterol (LDL) and serum triglycerides (Tg) were obtained from the Neale Lab (http://www.nealelab.is/uk-biobank). We used UK Biobank European ancestry individuals to calculate LD matrix and performed colocalization analysis with five different methods (Table 1) using prior colocalization probabilities varying from {$1\times 10^{-7}$, $1\times 10^{-6}$, $1\times 10^{-5}$, $1\times 10^{-4}$, $1\times 10^{-3}$}. In SharePro, the maximum number of effect groups was set to 10.

### 2.5 Colocalization analysis of RSPO3 protein QTL and eBMD GWAS

We examined the utility of SharePro by assessing the colocalization between a cis-protein QTL locus of the circulating abundance of RSPO3 and a GWAS locus identified for eBMD using heel quantitative ultrasound measurement. We obtained European ancestry-specific RSPO3 cis-protein QTL summary statistics from the Fenland study (Pietzner *et al.* 2021) and UK Biobank European ancestry-specific eBMD GWAS summary statistics from the GEFOS consortium (Morris *et al.* 2019). The LD matrix was calculated using UK Biobank European ancestry individuals and colocalization analysis was performed with five different methods (Table 1) using default prior settings. In SharePro, the maximum number of effect groups was set to 10.

## 3 Results

### 3.1 Simulation studies

**Figure 2.**
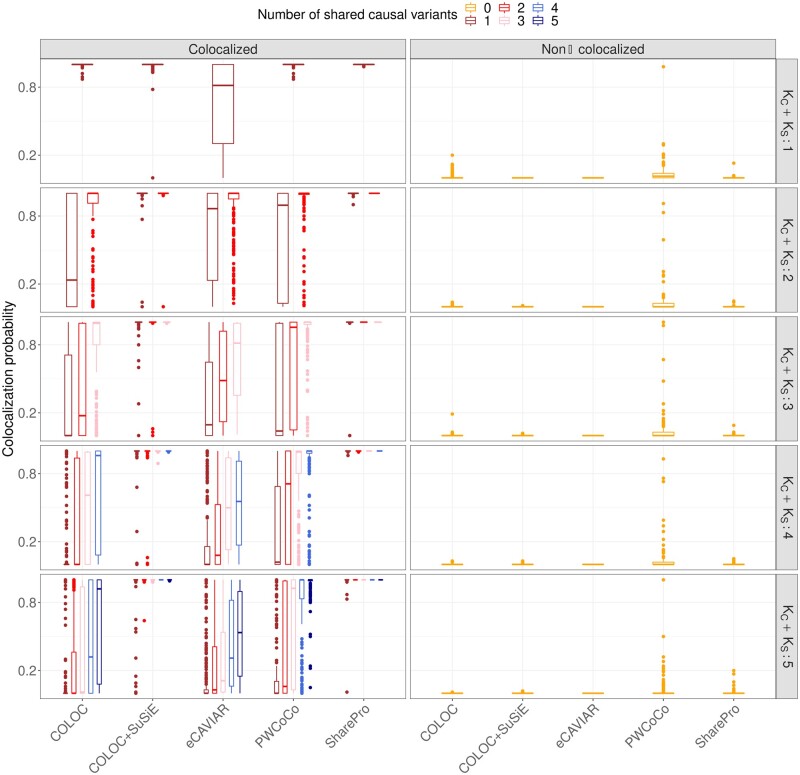
SharePro demonstrated improved power with a well-controlled false positive rate for colocalization analysis. Colocalization probabilities derived by 5 methods based on 50 replicates in each of the 5 loci are illustrated. Rows represent the different numbers of causal variants ($K_{C}+K_{S}$) and colors represent the different numbers of shared causal variants ($K_{C}$) between the two simulated traits. Median colocalization probabilities across a total of 250 replicates are indicated by horizontal bars and inter-quartile ranges are represented by boxes.

To evaluate the performance of SharePro in colocalization analysis, we performed simulations under different causal settings. SharePro achieved the highest power in most settings. Specifically, in the simple scenario of only one shared causal variant ($K_{C}=1$, $K_{S}=0$), COLOC, PWCoCo, and SharePro accurately identified all simulated cases of colocalization with a colocalization probability above 0.8 (Fig. 2 and Supplementary Table S1). Meanwhile, COLOC + SuSiE only identified 98.8% cases of colocalization (Supplementary Table S1) while the locus-level colocalization summary derived from eCAVIAR only identified 51.2% of the simulated cases of colocalization (Supplementary Table S1).

In more challenging scenarios with multiple causal variants, SharePro maintained the highest power for colocalization analysis, followed by COLOC + SuSiE. For example, with $K_{C}=1$ and $K_{S}=1$ and a colocalization probability cutoff at 0.8, SharePro successfully identified all of the simulated cases of colocalization, while the second best method COLOC + SuSiE achieved a true positive rate of 97.6% (Fig. 2 and Supplementary Table S1). In contrast, as expected, since the one-causal-variant assumption was not satisfied, the performance of COLOC became worse and only identified 44.4% of the simulated cases of colocalization (Supplementary Table S1). With more than one causal variant shared between the two simulated traits ($K_{C}\;>\;1$), SharePro consistently identified all cases of colocalization and outperformed other methods (Fig. 2 and Supplementary Table S1).

When causal variants were different across the simulated traits (noncolocalized), the colocalization probabilities obtained by COLOC, COLOC+SuSiE, eCAVIAR, and SharePro were consistently below 0.2, demonstrating well-controlled false positive rates. (Fig. 2 and Supplementary Table S2). However, PWCoCo generated higher colocalization probabilities in these simulated noncolocalized scenarios. For instance, when there were three causal variants for trait 1 and three different causal variants for trait 2 ($K_{C}=0$ and $K_{S}=3$), the colocalization probability generated by PWCoCo was <0.2 for 96.8% of the simulated cases of noncolocalization (Fig. 2 and Supplementary Table S2). However, PWCoCo incorrectly generated a colocalization probability >0.8 for 1.2% of these simulated cases of noncolocalization (Fig. 2 and Supplementary Table S1).

Moreover, SharePro also demonstrated high computational efficiency (Table 1). Across different simulation settings, on average, SharePro took 4.3 s to assess colocalization in a 1-Mb locus, which was only longer than COLOC. In contrast, on average, eCAVIAR took more than 3 minutes to assess colocalization in the same locus (Table 1).

**Figure 3.**
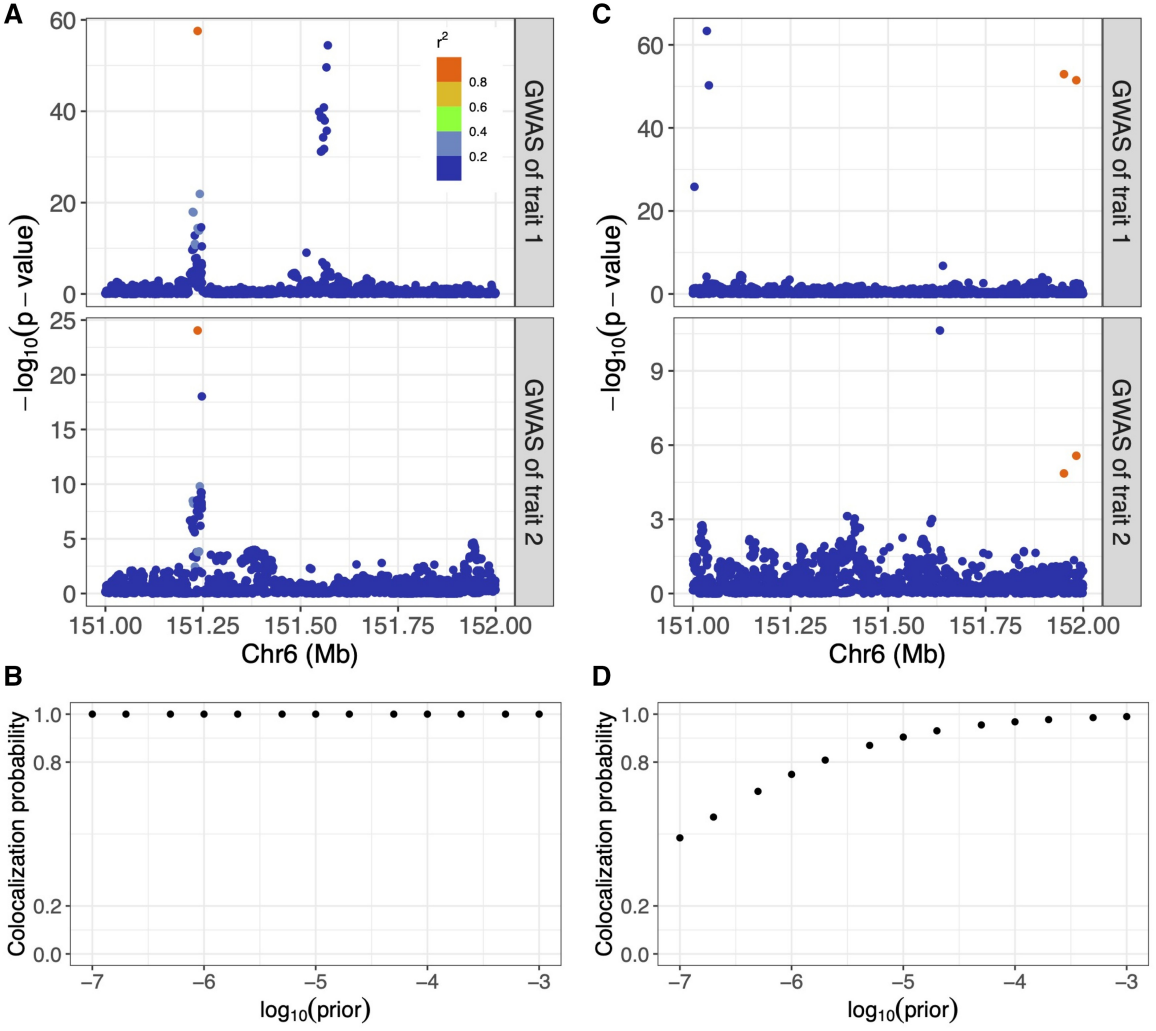
SharePro prior sensitivity analysis with two examples. (A) GWAS associations with a strong support for colocalization. Each dot represents a variant and the color indicates its correlation with the simulated colocalized variant. (B) Prior sensitivity analysis in the case of a strong support for colocalization. The *x*-axis stands for prior colocalization probabilities in the logarithmic scale and the *y*-axis stands for posterior colocalization probabilities. (C) GWAS associations with a weak support for colocalization. Each dot represents a variant and the color indicates its correlation with the simulated colocalized variant. (D) Prior sensitivity analysis in the case of a weak support for colocalization. The *x*-axis stands for prior colocalization probabilities in the logarithmic scale and the *y*-axis stands for posterior colocalization probabilities.

We additionally performed prior sensitivity analysis (Supplementary Note) to examine the impact of prior colocalization probabilities on colocalization results and showcased two representative scenarios in Fig. 3. When the GWAS summary statistics demonstrate a strong colocalization pattern (Fig. 3A), varying prior colocalization probabilities does not drastically change the posterior colocalization probabilities (Fig. 3B). In contrast, when the statistical evidence from GWAS associations is weak (Fig. 3C), the posterior colocalization probabilities increase with the prior colocalization probabilities (Fig. 3D). Additionally, increasing prior colocalization probability to $1\times 10^{-3}$ could lead to inflated posterior colocalization probabilities in some noncolocalized scenarios with only negligible improvement in colocalized scenarios for both COLOC and SharePro (Supplementary Fig. S1 and Supplementary Tables S3 and S4).

Furthermore, SharePro demonstrated the highest mean power in identifying individual shared causal variants across all simulation settings (Supplementary Fig. S2).

### 3.2 Colocalization analysis of cis-protein QTL of lipid-lowering drug target proteins and GWAS for lipid traits

**Figure 4.**
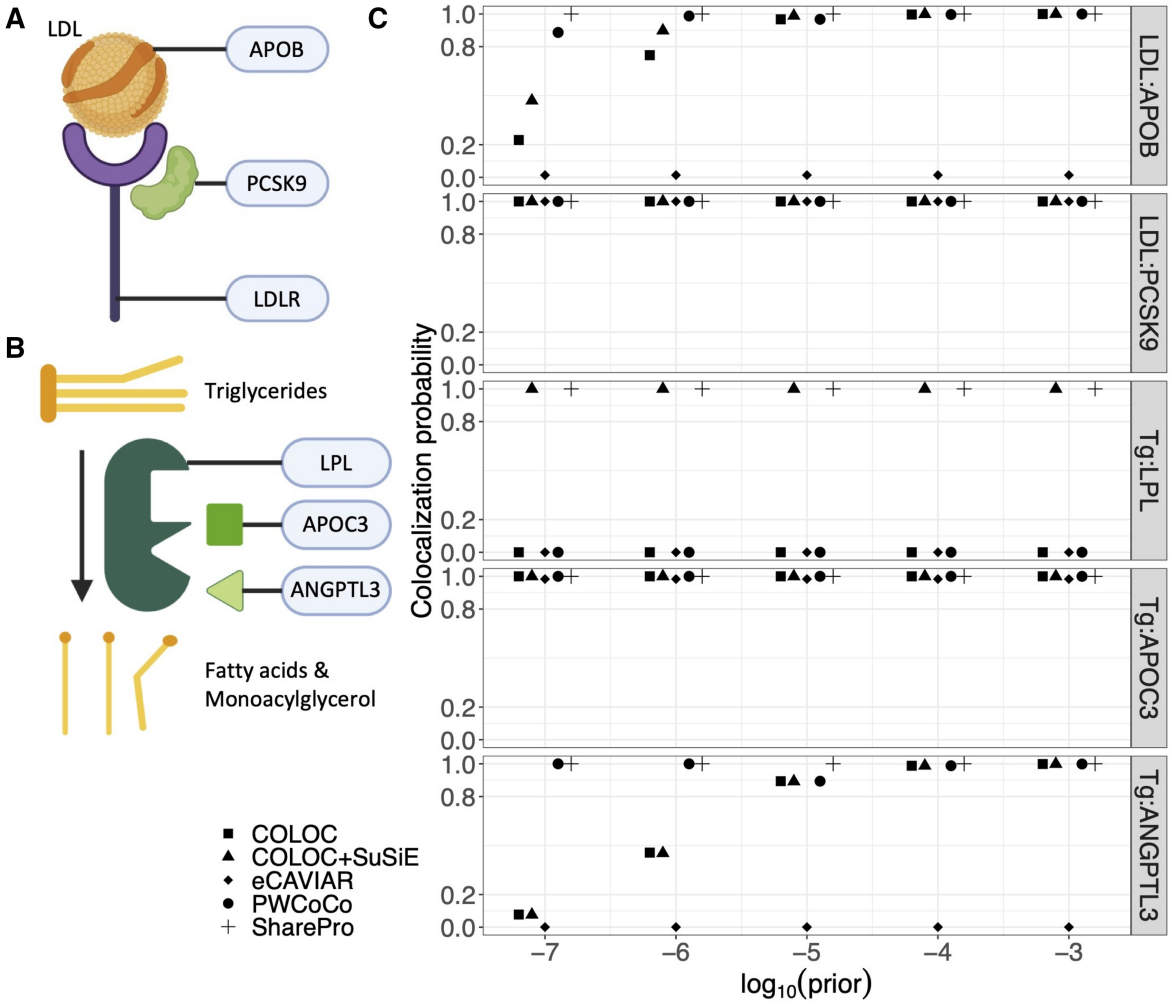
Colocalization analysis of cis-protein QTL of lipid-lowering drug target proteins and GWAS for lipid traits. (A) APOB and PCSK9 as targets for LDL-lowering drugs. APOB is the lipoprotein constituting LDL particles. PCSK9 promotes the degradation of LDL receptor (LDLR), which is essential for the uptake of LDL into cells. (B) LPL, APOC3, and ANGPTL3 as targets for Tg-lowering drugs. LPL catalyzes the hydrolysis of Tg, whose activity can be inhibited by APOC3 and ANGPTL3. (C) Colocalization probabilities for colocalization analysis of cis-protein QTL of lipid-lowering drug target proteins and GWAS for their corresponding lipid traits obtained from different colocalization methods under varying prior colocalization probabilities.

Lipid-lowering drugs that effectively decrease circulating LDL and Tg levels are important for improving and maintaining cardiovascular health for patients. Several lipid-lowering drugs act by modulating the circulating abundances of target proteins (Fig. 4A and B) (Li *et al.* 2023). Therefore, the colocalization analysis of cis-protein QTL of lipid-lowering drug target proteins and GWAS for the corresponding lipid traits can serve as useful positive controls for assessing the performance of colocalization methods.

APOB and PCSK9 are two important LDL-lowering drug target proteins (Li *et al.* 2023). Specifically, APOB is the primary structural protein of LDL particles (Sniderman *et al.* 2019), while PCSK9 is a key factor modulating lipid metabolism through its regulatory effect on LDL receptor (Stein *et al.* 2012) (Fig. 4A). In colocalization analysis of cis-protein QTL of APOB and LDL GWAS, SharePro and PWCoCo consistently identified the colocalized signal (Supplementary Fig. S3) with a colocalization probability >0.8 under varying prior colocalization probabilities, while the colocalization results obtained using COLOC and COLOC+SuSiE were sensitive to the choice of prior colocalization probability (Fig. 4C and Supplementary Table S5). Meanwhile, all colocalization methods provided colocalization evidence for cis-protein QTL of PCSK9 and LDL GWAS (Fig. 4C and Supplementary Table S5 and Supplementary Fig. S4).

Furthermore, LPL, APOC3, and ANGPTL3 are known Tg-lowering drug target proteins (Li *et al.* 2023). LPL is the essential enzyme catalyzing the hydrolysis of circulating Tg (Mead *et al.* 2002) (Fig. 4B). The activity of LPL can be inhibited by APOC3 and ANGPTL3 (Shimizugawa *et al.* 2002, TG, HDL Working Group of the Exome Sequencing Project National Heart Lung, and Blood Institute 2014) (Fig. 4B). SharePro and COLOC+SuSiE identified the colocalization of cis-protein QTL of LPL and Tg GWAS with a colocalization probability of 1.0 across all prior colocalization probabilities, while the other methods did not provide any colocalization evidence (Fig. 4C and Supplementary Table S5 and Supplementary Fig. S5).

All colocalization methods provided colocalization evidence for cis-protein QTL of APOC3 and Tg GWAS (Fig. 4C and Supplementary Table S5 and Supplementary Fig. S6). Moreover, only SharePro and PWCoCo were able to consistently identify the colocalization between cis-protein QTL of ANGPTL3 and Tg GWAS (Fig. 4C and Supplementary Table S5 and Supplementary Fig. S7).

### 3.3 RSPO3-eBMD example

The eBMD measured at the heel using quantitative ultrasound is an important biomarker of osteoporosis and strongly predicts fracture risk (Morris *et al.* 2019, Lu *et al.* 2021, 2022). RSPO3 is a known modulator of the Wnt signaling pathway that plays a crucial role in maintaining bone homeostasis (Baron and Kneissel 2013, Lerner and Ohlsson 2015). It has been experimentally shown that the abundance of RSPO3 strongly influences the proliferation and differentiation of osteoblasts and regulates bone mass (Nilsson *et al.* 2021). Therefore, it is biologically plausible that the cis-protein QTL of RSPO3 colocalize with an eBMD GWAS locus.

**Figure 5.**
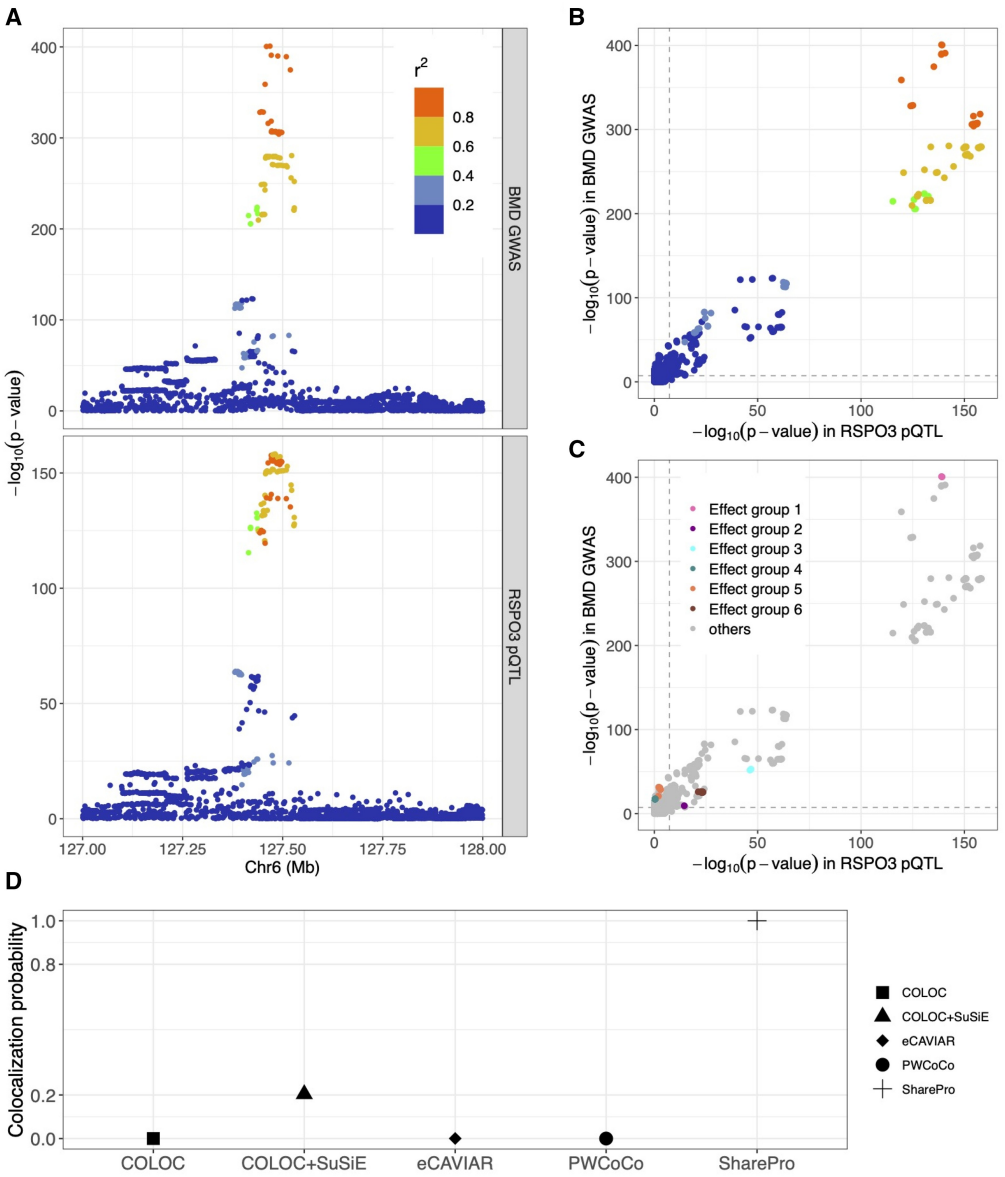
SharePro identified shared effect groups between RSPO3 cis-protein QTL and eBMD GWAS. (A) Marginal associations with eBMD GWAS and RSPO3 cis-protein QTL. The *x*-axis indicates chromosome and position information and the *y*-axis represents *P*-value on the logarithmic scale. Each dot represents one variant. Color indicates the correlation ($r^{2}$) between each variant and the colocalized variant rs7741021. (B) Scatterplot of marginal associations with RSPO3 cis-protein QTL and eBMD GWAS. Each dot represents one variant. Color indicates the correlation ($r^{2}$) between each variant and the colocalized variant. (C) Scatterplot of marginal associations for RSPO3 cis-protein QTL and eBMD GWAS. Each dot represents one variant. Color indicates the effect group that each variant belongs to. (D) Colocalization probabilities assessed by different colocalization methods for RSPO3 cis-protein QTL and eBMD GWAS.

The marginal genetic associations with RSPO3 abundance and eBMD are illustrated in Fig. 5A and B. With SharePro, we identified multiple effect groups in this region and colocalization results indicated that both rs7741021/rs9482773 and rs853974 were shared causal signals between circulating RSPO3 abundance and eBMD (Fig. 5C and Supplementary Table S6). Interestingly, although these genetic associations demonstrated a highly similar pattern (Fig. 5A and B), existing methods indicated no or minor evidence of colocalization (Fig. 5D). We explored different hyperparameter settings for prior colocalization probabilities in SharePro (Supplementary Note) and obtained robust colocalization results (Supplementary Tables S7–S10).

## 4 Discussion

In this work, we present SharePro to integrate LD modeling and colocalization assessment that extends the classical COLOC framework to account for multiple causal signals. Compared to methods that adopt a two-step strategy to relax the one-causal-variant assumption in COLOC, the effect group-level approach in SharePro can effectively reduce the impact of LD in aligning causal signals, resulting in improved power for colocalization analysis.

Under different simulation settings, SharePro achieved the highest power with a well-controlled false positive rate. Notably, when there was only one causal variant, which satisfies the one-causal-variant assumption of COLOC, the power of SharePro was comparable to that of COLOC. With multiple causal variants, when most of the causal variants were shared between the two traits, SharePro and COLOC+SuSiE demonstrated similar performance. Nonetheless, SharePro achieved higher power than COLOC+SuSiE if the proportion of causal variants being shared is low. Additionally, SharePro also demonstrated high computational efficiency.

An important hyperparameter in colocalization analysis is the prior colocalization probability. In SharePro, the default prior colocalization probability is $1\;\times \;10^{-5}$. In COLOC, this hyperparameter is represented as $p_{12}$ with a default value of $1\times 10^{-5}$ (Giambartolomei *et al.* 2014). We demonstrated that using a large prior colocalization probability could potentially increase the false positive rate. In our simulation studies, increasing prior colocalization probability to $1\times 10^{-3}$ resulted in inflated posterior colocalization probabilities in some noncolocalized scenarios for both COLOC and SharePro. Therefore, we believe that extra caution should be taken in using a high prior colocalization probability in colocalization analysis. In practice, it can also be informative to explore a different range of values to evaluate the robustness of colocalization results with respect to the prior colocalization probability, especially when GWAS are not well-powered (Wallace 2020). Future investigations based on real data are warranted to more comprehensively compare the utility of such sensitivity analyses versus pre-specifying the prior colocalization probability, such as using ENLOC/fastENLOC (Wen *et al.* 2017, Hukku *et al.* 2021).

Since SharePro assesses colocalization at the effect group level, it has the ability to identify individual colocalized signals. In our simulation analysis, compared to COLOC+SuSiE and eCAVIAR, SharePro demonstrated the highest power in identifying specific shared causal variants. This feature can be useful in characterizing the local genetic architecture of various complex traits.

We showcased the power of SharePro in real data analyses using positive controls. Based on circulating protein QTL studies for five well-established lipid-lowering drug target proteins and GWAS for lipid traits, we demonstrated that SharePro was the only method that consistently yielded high colocalization probabilities for all pairs of drug target proteins and lipid traits across varying prior colocalization probabilities.

With the colocalization analysis of RSPO3 cis-protein QTL and eBMD GWAS, we demonstrated that SharePro could correctly identify biologically plausible colocalization in the presence of multiple causal signals. In the RSPO3 locus, both the RSPO3 protein QTL study and the eBMD GWAS are well-powered and the marginal associations exhibit a similar pattern. However, the lead variants with the smallest marginal p-value in this locus, although highly correlated, are different for circulating RSPO3 abundance and eBMD. In the presence of multiple causal signals, colocalization analysis in this locus using existing methods has been challenging. In SharePro, correlated variants are grouped into effect groups and as a result, the impact of misaligned lead variants on colocalization analysis is mitigated.

Colocalization analysis shares some similar features with cross-population fine-mapping, although these analyses have different purposes. Colocalization analysis is primarily used to contrast the genetic signals of different traits while in cross-population fine-mapping, the main goal is to aggregate genetic signals for the same trait in different populations. Therefore, compared to cross-population fine-mapping methods (Cai *et al.* 2023, Yuan *et al.* 2023) that assume causal signals are shared across populations, a unique feature in the SharePro model is the introduction of trait-specific causal indicators for modeling trait-specific causal signals. Our recent work (Zhang *et al.* 2023b) demonstrated that SharePro can be adapted to account for genetic heterogeneity across sub-populations with different environmental exposures. The potential of further adapting SharePro for improved cross-population fine-mapping may also warrant exploration.

There are other cautions in colocalization analysis that also apply to SharePro. First, users are strongly recommended to perform summary statistics-based analyses using an LD reference panel that matches the LD structure underlying the samples included in GWAS. In SharePro, severe LD mismatch can lead to convergence issues of the algorithm. Second, the validity of colocalization results relies on the rigor of GWAS in carefully accounting for population stratification and other unmeasured confounding factors, since variants associated with shared confounding factors can also be considered colocalized. Third, the power to detect colocalization is dependent on the power of GWAS. We strongly suggest that well-powered GWAS should be used for colocalization analysis.

In summary, we have developed SparsePro to extend COLOC for colocalization analysis. With increased power and a well-controlled false positive rate at a low computation cost, SharePro demonstrated great utility in assessing whether two or more traits share the same genetic signals. With the increasing number of publicly available GWAS summary statistics, we envision SharePro will have the potential to substantially deepen our understanding of complex traits and diseases.

## Supplementary Material

btae295_Supplementary_Data

## Data and availability

The SharePro software for colocalization analysis is openly available at https://github.com/zhwm/SharePro_coloc and the analysis conducted in this study is available at https://github.com/zhwm/SharePro_coloc_analysis. GWAS summary statistics for LDL and Tg was obtained from the Neale lab at http://www.nealelab.is/uk-biobank. GWAS summary statistics for eBMD was obtained from the GEFOS consortium at http://www.gefos.org. GWAS summary statistics for cis-protein QTL was obtained from https://omicscience.org/apps/pgwas/ or http://ukb-ppp.gwas.eu. Both COLOC and COLOC+SuSiE are included in the coloc (version 5.1.0) R package obtained from CRAN. PWCoCo was obtained from GitHub at https://github.com/jwr-git/pwcoco. eCAVIAR was obtained from GitHub at https://github.com/fhormoz/caviar.
