# Supplementary material for: SharePro: an accurate and efficient genetic colocalization method accounting for multiple causal signals
Source: Bioinformatics. 2024 Apr 30;40(5):btae295. doi: 10.1093/bioinformatics/btae295 (PMC11105950; doi:10.1093/bioinformatics/btae295)
Supplement: btae295_Supplementary_Data [file btae295_supplementary_data.zip › note.pdf]

# SharePro for colocalization Supplementary Note

Wenmin Zhang[1,2,*], Tianyuan Lu[3], Robert Sladek[1,4,5], Yue Li[1,6], Hamed S. Najafabadi[1,4,5], and Josée Dupuis[1,7,*]

[1]Quantitative Life Sciences, McGill University, Montreal, Canada
[2]Montreal Heart Institute, Montreal, Canada
[3]Department of Statistical Sciences, University of Toronto, Toronto, Canada
[4]Department of Human Genetics, McGill University, Montreal, Canada
[5]Dahdaleh Institute of Genomic Medicine, Montreal, Canada
[6]School of Computer Science, McGill University, Montreal, Canada
[7]Department of Epidemiology, Biostatistics and Occupational Health, McGill University, Montreal, Canada
[*]Correspondence to wenmin.zhang@mail.mcgill.ca and josee.dupuis3@mcgill.ca

## 1 A variational inference algorithm for Bayesian colocalization

In SharePro, similar to our previous work on the sparse projection formulation of the SuSiE model (Zhang et al., 2023; Wang et al., 2020; Zou et al., 2022), with a shared projection matrix $\mathbf{S}_{G\times K} = [\mathbf{s}_1, ..., \mathbf{s}_K]$, we can group correlated variants into $K$ effect groups, where

$$\mathbf{s}_k \sim \textbf{Multinomial}(1, \mathbf{1}_{G\times 1} \times \frac{1}{G})$$

is the sparse indicator for the variant compositions in the $k^{th}$ effect group. We have trait-specific indicator vectors $\mathbf{c}_1 = [c_{11}, ..., c_{K1}]$ and $\mathbf{c}_2 = [c_{12}, ..., c_{K2}]$ to characterize the causal statuses of effect groups in each trait where

$$c_{k1}, c_{k2} \sim \textbf{Bernoulli}(\sigma)$$

With trait-specific effect size vectors $\boldsymbol{\beta}_1 = [\beta_{11}, ..., \beta_{K1}]$ and $\boldsymbol{\beta}_2 = [\beta_{12}, ..., \beta_{K2}]$, where

$$\beta_{k1} \sim \mathcal{N}(0, \tau_{\beta_1}^{-1})$$

$$\beta_{k2} \sim \mathcal{N}(0, \tau_{\beta_2}^{-1})$$

and denoting the genotype matrix as $\mathbf{X}_1$ and $\mathbf{X}_2$, for traits $\mathbf{y}_1$ and $\mathbf{y}_2$, we have:

$$\mathbf{y}_1 \sim \mathcal{N}(\mathbf{X}_1 \sum_k \mathbf{s}_k \beta_{k1} c_{k1}, \tau_{y_1}^{-1}\mathbf{I})$$

$$\mathbf{y}_2 \sim \mathcal{N}(\mathbf{X}_2 \sum_k \mathbf{s}_k \beta_{k2} c_{k2}, \tau_{y_2}^{-1}\mathbf{I})$$

In colocalization analysis, we are interested in the posterior probabilities of causal indicators based on the observed traits $\mathbf{y}_1$, $\mathbf{y}_2$ and the genotypes $\mathbf{X}_1$ and $\mathbf{X}_2$. Inference of the exact posterior distribution of causal indicators $\mathbf{c}_1$, $\mathbf{c}_2$ and variant representations in effect groups $\mathbf{S}$ is difficult. Similar to the iterative Bayesian stepwise selection (IBSS) algorithm (Wang et al., 2020) proposed in SuSiE and our previous work (Zhang et al., 2023), we use a paired mean field factorized variational family (Titsias and Lazaro-Gredilla, 2011)

$$q(\mathbf{S}, \boldsymbol{\beta}_1, \boldsymbol{\beta}_2, \mathbf{c}_1, \mathbf{c}_2) = \prod_k q(\mathbf{s}_k, \beta_{k1}, \beta_{k2}, c_{k1}, c_{k2})$$

to approximate the desired posterior distribution:

$$p(\mathbf{S}, \boldsymbol{\beta}_1, \boldsymbol{\beta}_2, \mathbf{c}_1, \mathbf{c}_2 | \mathbf{y}_1, \mathbf{y}_2, \mathbf{X}_1, \mathbf{X}_2) = \frac{p(\mathbf{y}_1, \mathbf{y}_2, \mathbf{S}, \boldsymbol{\beta}_1, \boldsymbol{\beta}_2, \mathbf{c}_1, \mathbf{c}_2 | \mathbf{X}_1, \mathbf{X}_2)}{p(\mathbf{y}_1, \mathbf{y}_2 | \mathbf{X}_1, \mathbf{X}_2)}$$

We can obtain the optimal approximation by maximizing the evidence lower bound (ELBO) (Blei et al., 2017):

$$ELBO = E_{q(\mathbf{S}, \boldsymbol{\beta}_1, \boldsymbol{\beta}_2, \mathbf{c}_1, \mathbf{c}_2)}[\log \frac{p(\mathbf{y}_1, \mathbf{y}_2, \mathbf{S}, \boldsymbol{\beta}_1, \boldsymbol{\beta}_2, \mathbf{c}_1, \mathbf{c}_2 | \mathbf{X}_1, \mathbf{X}_2)}{q(\mathbf{S}, \boldsymbol{\beta}_1, \boldsymbol{\beta}_2, \mathbf{c}_1, \mathbf{c}_2)}]$$

with the following conditions satisfied for each $k$ (Blei et al., 2017):

$$\log q(\mathbf{s}_k, \beta_{k1}, \beta_{k2}, c_{k1}, c_{k2}) = E_{q(\mathbf{S}_{\backslash k}, \boldsymbol{\beta}_{\backslash k1}, \boldsymbol{\beta}_{\backslash k2}, \mathbf{c}_{\backslash k1}, \mathbf{c}_{\backslash k2})} [\log p(\mathbf{y}_1, \mathbf{y}_2, \mathbf{S}, \boldsymbol{\beta}_1, \boldsymbol{\beta}_2, \mathbf{c}_1, \mathbf{c}_2 | \mathbf{X}_1, \mathbf{X}_2)]$$

where $E_{q(\mathbf{S}_{\backslash k}, \boldsymbol{\beta}_{\backslash k1}, \boldsymbol{\beta}_{\backslash k2}, \mathbf{c}_{\backslash k1}, \mathbf{c}_{\backslash k2})}$ is the expectation with respect to the variational distribution excluding the $k^{th}$ component. If we write out the joint probability:

$$\begin{aligned}
&\log p(\mathbf{y}_1, \mathbf{y}_2, \mathbf{S}, \boldsymbol{\beta}_1, \boldsymbol{\beta}_2, \mathbf{c}_1, \mathbf{c}_2 | \mathbf{X}_1, \mathbf{X}_2) \\
=&\log p(\mathbf{y}_1 | \mathbf{X}_1, \mathbf{S}, \boldsymbol{\beta}_1, \mathbf{c}_1) + \log p(\mathbf{y}_2 | \mathbf{X}_2, \mathbf{S}, \boldsymbol{\beta}_2, \mathbf{c}_2) + \sum_k \log p(\mathbf{s}_k) \\
&+ \sum_k \log p(\beta_{k1}) + \sum_k \log p(\beta_{k2}) + \sum_k \log p(c_{k1}) + \sum_k \log p(c_{k2}) \\
=&\frac{N}{2} \log \frac{\tau_{y_1}}{2\pi} - \frac{\tau_{y_1}}{2} (\mathbf{y}_1 - \mathbf{X}_1 (\sum_k \mathbf{s}_k \beta_{k1} c_{k1}))^\top (\mathbf{y}_1 - \mathbf{X}_1 (\sum_k \mathbf{s}_k \beta_{k1} c_{k1})) \\
&+ \frac{N}{2} \log \frac{\tau_{y_2}}{2\pi} - \frac{\tau_{y_2}}{2} (\mathbf{y}_2 - \mathbf{X}_2 (\sum_k \mathbf{s}_k \beta_{k2} c_{k2}))^\top (\mathbf{y}_2 - \mathbf{X}_2 (\sum_k \mathbf{s}_k \beta_{k2} c_{k2})) \\
&+ \sum_k \sum_g s_{kg} \log \frac{1}{G} + \sum_k (\frac{1}{2} \log \frac{\tau_{\beta_1}}{2\pi} - \frac{\tau_{\beta_1}}{2} \beta_{k1}^2) + \sum_k (\frac{1}{2} \log \frac{\tau_{\beta_2}}{2\pi} - \frac{\tau_{\beta_2}}{2} \beta_{k2}^2) \\
&+ \sum_k [c_{k1} \log \sigma + (1 - c_{k1}) \log(1 - \sigma)] + \sum_k [c_{k2} \log \sigma + (1 - c_{k2}) \log(1 - \sigma)]
\end{aligned}$$

and denoting $\tilde{\boldsymbol{\beta}}_{\backslash k1} = E_{q(\mathbf{S}_{\backslash k}, \boldsymbol{\beta}_{\backslash k1}, \mathbf{c}_{\backslash k1})} \left[\sum_{k' \neq k} \mathbf{s}_{k'} \beta_{k'1} c_{k'1}\right]$ and $\tilde{\boldsymbol{\beta}}_{\backslash k2} = E_{q(\mathbf{S}_{\backslash k}, \boldsymbol{\beta}_{\backslash k2}, \mathbf{c}_{\backslash k2})} \left[\sum_{k' \neq k} \mathbf{s}_{k'} \beta_{k'2} c_{k'2}\right]$ the required conditions can be simplified into four different cases for the $k^{th}$ effect group:

**Case 1**: $c_{k1} = 0 = c_{k2} = 0$, i.e. the $k^{th}$ effect group is not causal for either trait:

$$\begin{aligned}
&\log q(s_{kg} = 1, \mathbf{s}_{k \backslash g} = \mathbf{0}, \beta_{k1}, \beta_{k2}, c_{k1} = 0, c_{k2} = 0) \\
=&const + \frac{1}{2} \log \frac{\tau_{\beta_1}}{2\pi} - \frac{\tau_{\beta_1}}{2} \beta_{k1}^2 + \frac{1}{2} \log \frac{\tau_{\beta_2}}{2\pi} - \frac{\tau_{\beta_2}}{2} \beta_{k2}^2 + 2 \log(1 - \sigma)
\end{aligned}$$

After integrating out $\beta_{k1}$ and $\beta_{k2}$, we have:

$$\log q(s_{kg}=1, \mathbf{s}_{k\backslash g}=\mathbf{0}, c_{k1}=0, c_{k2}=0) = const + 2\log(1-\sigma)$$

**Case 2 (trait 1 specific)**: $c_{k1}=1$ and $c_{k2}=0$, i.e. the $k^{th}$ effect group is causal for trait $\mathbf{y}_1$ but not for trait $\mathbf{y}_2$:

$$\begin{aligned}
&\log q(s_{kg}=1, \mathbf{s}_{k\backslash g}=\mathbf{0}, \beta_{k1}, \beta_{k2}, c_{k1}=1, c_{k2}=0) \\
=&const + \tau_{y_1}\mathbf{X}_{g1}^\top(\mathbf{y_1} - \mathbf{X}_1\tilde{\boldsymbol{\beta}}_{\backslash k1})\beta_{k1} - \frac{\tau_{y_1}}{2}\mathbf{X}_{g1}^\top\mathbf{X}_{g1}\beta_{k1}^2 + \frac{1}{2}\log\frac{\tau_{\beta_1}}{2\pi} - \frac{\tau_{\beta_1}}{2}\beta_{k1}^2 \\
&+ \frac{1}{2}\log\frac{\tau_{\beta_2}}{2\pi} - \frac{\tau_{\beta_2}}{2}\beta_2^2 + \log\sigma + \log(1-\sigma)
\end{aligned}$$

We recognize that $q(\beta_{k1}|s_{kg=1}, \mathbf{s}_{k\backslash g}=\mathbf{0}, c_{k1}=1, c_{k2}=0) \sim \mathcal{N}(\mu_{kg1}^*, \tau_{kg1}^*)$. By matching sufficient statistics of the normal distribution, we have variational parameters for $\beta_{k1}$:

$$\tau_{kg1}^* = \tau_{y_1}\mathbf{X}_{g1}^\top\mathbf{X}_{g1} + \tau_{\beta_1}$$

$$\mu_{kg1}^* = \frac{\tau_{y_1}}{\tau_{kg1}^*}\mathbf{X}_{g1}^\top(\mathbf{y_1} - \mathbf{X}_1\tilde{\boldsymbol{\beta}}_{\backslash k1})$$

After integrating out $\beta_{k1}$ and $\beta_{k2}$, we have:

$$\log q(s_{kg}=1, \mathbf{s}_{k\backslash g}=\mathbf{0}, c_{k1}=1, c_{k2}=0) = const + \frac{1}{2}\log\frac{\tau_{\beta_1}}{\tau_{kg1}^*} + \frac{\tau_{kg1}^*\mu_{kg1}^{*2}}{2} + \log\sigma(1-\sigma)$$

**Case 3 (trait 2 specific)**: $c_{k1}=0$ and $c_{k2}=1$, i.e. the $k^{th}$ effect group is causal for trait $\mathbf{y}_2$ but not for trait $\mathbf{y}_1$:

$$\begin{aligned}
&\log q(s_{kg}=1, \mathbf{s}_{k\backslash g}=\mathbf{0}, \beta_{k1}, \beta_{k2}, c_{k1}=0, c_{k2}=1) \\
=&const + \tau_{y_2}\mathbf{X}_{g2}^\top(\mathbf{y_2} - \mathbf{X}_2\tilde{\boldsymbol{\beta}}_{\backslash k2})\beta_{k2} - \frac{\tau_{y_2}}{2}\mathbf{X}_{g2}^\top\mathbf{X}_{g2}\beta_{k2}^2 + \frac{1}{2}\log\frac{\tau_{\beta_1}}{2\pi} - \frac{\tau_{\beta_1}}{2}\beta_{k1}^2 \\
&+ \frac{1}{2}\log\frac{\tau_{\beta_2}}{2\pi} - \frac{\tau_{\beta_2}}{2}\beta_{k2}^2 + \log\sigma + \log(1-\sigma)
\end{aligned}$$

Similarly, we recognize that $q(\beta_{k2}|s_{kg=1}, \mathbf{s}_{k\backslash g}=\mathbf{0}, c_{k1}=0, c_{k2}=1) \sim \mathcal{N}(\mu_{kg2}^*, \tau_{kg2}^*)$. By matching sufficient statistics for the normal distribution, we can obtain the following variational parameters for $\beta_{k2}$:

$$\tau_{kg2}^* = \tau_{y_2}\mathbf{X}_{g2}^\top\mathbf{X}_{g2} + \tau_{\beta_2}$$

$$\mu_{kg2}^* = \frac{\tau_{y_2}}{\tau_{kg2}^*}\mathbf{X}_{g2}^\top(\mathbf{y_2} - \mathbf{X}_2\tilde{\boldsymbol{\beta}}_{\backslash k2})$$

After integrating out $\beta_{k1}$ and $\beta_{k2}$, we have:

$$\log q(s_{kg}=1, \mathbf{s}_{k\backslash g}=\mathbf{0}, c_{k1}=0, c_{k2}=1) = const + \frac{1}{2}\log\frac{\tau_{\beta_2}}{\tau_{kg2}^*} + \frac{\tau_{kg2}^*\mu_{kg2}^{*2}}{2} + \log\sigma(1-\sigma)$$

**Case 4 (colocalization)**: $c_{k1}=1=c_{k2}=1$, i.e. the $k^{th}$ effect group is causal for both trait $\mathbf{y}_1$ and trait $\mathbf{y}_2$:

$$\begin{aligned}
&\log q(s_{kg}=1, \mathbf{s}_{k\backslash g}=\mathbf{0}, \beta_{k1}, \beta_{k2}, c_{k1}=1, c_{k2}=1) \\
=&const + \tau_{y_1}\mathbf{X}_{g1}^\top(\mathbf{y_1} - \mathbf{X}_1\tilde{\boldsymbol{\beta}}_{\backslash k1})\beta_{k1} - \frac{\tau_{y_1}}{2}\mathbf{X}_{g1}^\top\mathbf{X}_{g1}\beta_{k1}^2 + \tau_{y_2}\mathbf{X}_{g2}^\top(\mathbf{y_2} - \mathbf{X}_2\tilde{\boldsymbol{\beta}}_{\backslash k2})\beta_{k2} \\
&- \frac{\tau_{y_2}}{2}\mathbf{X}_{g2}^\top\mathbf{X}_{g2}\beta_{k2}^2 + \frac{1}{2}\log\frac{\tau_{\beta_1}}{2\pi} - \frac{\tau_{\beta_1}}{2}\beta_{k1}^2 + \frac{1}{2}\log\frac{\tau_{\beta_2}}{2\pi} - \frac{\tau_{\beta_2}}{2}\beta_{k2}^2 + 2\log\sigma
\end{aligned}$$

We recognize that $q(\beta_{k1}|s_{kg=1}, \mathbf{s}_{k\backslash g} = \mathbf{0}, c_{k1} = 1, c_{k2} = 1) \sim \mathcal{N}(\mu^*_{kg1}, \tau^*_{kg1})$ and $q(\beta_{k2}|s_{kg=1}, \mathbf{s}_{k\backslash g} = \mathbf{0}, c_{k1} = 1, c_{k2} = 1) \sim \mathcal{N}(\mu^*_{kg2}, \tau^*_{kg2})$. By matching sufficient statistics for these normal distribution, we obtain the following variational parameters for $\beta_{k1}$ and $\beta_{k2}$:

$$
\begin{aligned}
\tau^*_{kg1} &= \tau_{y_1}\mathbf{X}_{g1}^\top\mathbf{X}_{g1} + \tau_{\beta_1} \\
\mu^*_{kg1} &= \frac{\tau_{y_1}}{\tau^*_{kg1}}\mathbf{X}_{g1}^\top(\mathbf{y_1} - \mathbf{X}_1\tilde{\boldsymbol{\beta}}_{\backslash k1}) \\
\tau^*_{kg2} &= \tau_{y_2}\mathbf{X}_{g2}^\top\mathbf{X}_{g2} + \tau_{\beta_2} \\
\mu^*_{kg2} &= \frac{\tau_{y_2}}{\tau^*_{kg2}}\mathbf{X}_{g2}^\top(\mathbf{y_2} - \mathbf{X}_2\tilde{\boldsymbol{\beta}}_{\backslash k2})
\end{aligned}
$$

After integrating out $\beta_{k1}$ and $\beta_{k2}$, we have:

$$
\begin{aligned}
\log q(s_{kg} = 1, \mathbf{s}_{k\backslash g} = \mathbf{0}, c_{k1} = 1, c_{k2} = 1) = \mathit{const} + \frac{1}{2}\log\frac{\tau_{\beta_1}}{\tau^*_{kg1}} + \frac{\tau^*_{kg1}\mu^{*2}_{kg1}}{2} \\
+ \frac{1}{2}\log\frac{\tau_{\beta_2}}{\tau^*_{kg2}} + \frac{\tau^*_{kg2}\mu^{*2}_{kg2}}{2} + 2\log\sigma
\end{aligned}
$$

Combining all four cases, we have the conditional distributions for $c_{k1}$ and $c_{k2}$:

$$
\begin{aligned}
q(c_{k1} = 1|s_{kg} = 1, \mathbf{s}_{k\backslash g} = \mathbf{0}) &= \frac{1}{1 + e^{-u_1}} \\
q(c_{k2} = 1|s_{kg} = 1, \mathbf{s}_{k\backslash g} = \mathbf{0}) &= \frac{1}{1 + e^{-u_2}}
\end{aligned}
$$

where

$$
\begin{aligned}
u_1 &= \frac{1}{2}\log\frac{\tau_{\beta_1}}{\tau^*_{kg1}} + \frac{\tau^*_{kg1}\mu^{*2}_{kg1}}{2} + \log\frac{\sigma}{1-\sigma} \\
u_2 &= \frac{1}{2}\log\frac{\tau_{\beta_2}}{\tau^*_{kg2}} + \frac{\tau^*_{kg2}\mu^{*2}_{kg2}}{2} + \log\frac{\sigma}{1-\sigma}
\end{aligned}
$$

After integrating out $c_{k1}$ and $c_{k2}$, we have the variational distribution for $\mathbf{s}_k$:

$$
\log q(s_{kg} = 1, \mathbf{s}_{k\backslash g} = \mathbf{0}) = \log\tilde{\pi}_g + 2\log(1-\sigma) + \log(1 + e^{u_1}) + \log(1 + e^{u_2})
$$

Therefore, for the $k^{th}$ effect groups, we can calculate the posterior colocalization probability as

$$
\begin{aligned}
&p(c_{k1} = c_{k2} = 1|\mathbf{y}_1, \mathbf{y}_2, \mathbf{X}_1, \mathbf{X}_2) \\
=&\sum_g q(c_{k1} = 1|s_{kg} = 1, \mathbf{s}_{k\backslash g} = \mathbf{0})q(c_{k2} = 1|s_{kg} = 1, \mathbf{s}_{k\backslash g} = \mathbf{0})q(s_{kg} = 1, \mathbf{s}_{k\backslash g} = \mathbf{0})
\end{aligned}
$$

In summary, we have now derived **Algorithm** 1 for colocalization analysis with SharePro:

**Algorithm 1:** SharePro for genetic colocalization analysis

**Data:** $\mathbf{X}_1^T\mathbf{X}_1$, $\mathbf{X}_2^T\mathbf{X}_2$, $\mathbf{X}_1^T\mathbf{y}_1$ and $\mathbf{X}_2^T\mathbf{y}_2$;
hyperparameters $\sigma$, $\tau_{\beta_1}$, $\tau_{\beta_2}$, $\tau_{y_1}$ and $\tau_{y_2}$
**Result:** Posterior colocalization probabilities for the $k^{th}$ effect group, $k \in \{1, ..., K\}$
**while** *ELBO not converge* **do**
  **for** $k = 1$ **to** $K$ **do**
    update $q(\mathbf{s}_k)$;
    update $q(c_{k1}|\mathbf{s}_k)$ and $q(c_{k2}|\mathbf{s}_k)$;
    update $q(\beta_{k1}|c_{k1}, \mathbf{s}_k)$ and $q(\beta_{k2}|c_{k2}, \mathbf{s}_k)$;
  **end**
**end**
**for** $k = 1$ **to** $K$ **do**
  $p(c_{k1} = c_{k2} = 1|\mathbf{y}_1, \mathbf{y}_2, \mathbf{X}_1, \mathbf{X}_2) = \sum_{\mathbf{s}_k} q(c_{k1} = 1|\mathbf{s}_k)q(c_{k2} = 1|\mathbf{s}_k)q(\mathbf{s}_k)$
**end**

# 2 Adaptation to summary statistics

The information in individual-level data $\mathbf{X}_1$, $\mathbf{X}_2$, $\mathbf{y}_1$ and $\mathbf{y}_2$ are used in the form of $\mathbf{X}_1^T\mathbf{X}_1$, $\mathbf{X}_2^T\mathbf{X}_2$, $\mathbf{X}_1^T\mathbf{y}_1$ and $\mathbf{X}_2^T\mathbf{y}_2$ throughout the proposed **Algorithm** 1, which can be derived from GWAS summary statistics and a LD reference panel. Specifically, in most publicly available GWAS summary statistics, GWAS z-scores are usually available or can be derived from marginal effect sizes and standard errors. With standardized genotypes and phenotypes, we have:

$$\mathbf{X}_1^\top\mathbf{X}_1 = N_1 * \mathbf{LD}$$

$$\mathbf{X}_2^\top\mathbf{X}_2 = N_2 * \mathbf{LD}$$

$$\mathbf{X}_1^\top\mathbf{y}_1 = \sqrt{N_1}\mathbf{z_1}$$

$$\mathbf{X}_2^\top\mathbf{y}_2 = \sqrt{N_2}\mathbf{z_2}$$

where $N_1$ and $N_2$ are sample sizes, $\mathbf{LD}$ is the variant Pearson correlation coefficient matrix and $\mathbf{z}_1$ and $\mathbf{z}_2$ are the z-scores in GWAS summary statistics for trait 1 and trait 2 respectively.

# 3 Hyperparameter estimation

Apart from the required quantities derived from GWAS summary statistics, there are also hyperparameters to be estimated in the colocalization algorithm: $\tau_{\beta_1}$ and $\tau_{\beta_2}$ in effect size distributions, $\tau_{y_1}$ and $\tau_{y_2}$ in trait distributions and $\sigma$ in the distributions of causal indicators. As shown in our previous work (Zhang et al., 2023), HESS-based heritability estimates (Shi et al., 2016) can provide suitable estimation for hyperparameters. Specifically, we can obtain the local heritability ($\hat{h}^2$) in a locus as well as per-variant heritability ($\hat{h}_v^2$) with the HESS (Shi et al., 2016) estimator using GWAS summary statistics, and use them to set hyperparameters: $\tau_{\beta_1}^{-1} = \hat{h}_{v1}^2$, $\tau_{\beta_2}^{-1} = \hat{h}_{v2}^2$, $\tau_{y_1}^{-1} = 1 - \hat{h}_1^2$ and $\tau_{y_2}^{-1} = 1 - \hat{h}_2^2$.

An important hyperparameter in Bayesian colocalization is the prior colocalization probability. We set its default value to $1 \times 10^{-5}$ (the same default value as used in COLOC). However, the impact of prior colocalization probabilities on posterior colocalization probabilities depends on the

power of GWAS. In simulation studies, we explored a range of prior: $1 \times 10^{-7}$, $2 \times 10^{-7}$, $5 \times 10^{-7}$, $1 \times 10^{-6}$, $2 \times 10^{-6}$, $5 \times 10^{-6}$, $1 \times 10^{-5}$, $2 \times 10^{-5}$, $5 \times 10^{-5}$, $1 \times 10^{-4}$, $2 \times 10^{-4}$, $5 \times 10^{-4}$, $1 \times 10^{-3}$ and showcased two representative simulation examples in **Figure 3**.

# References


David M Blei, Alp Kucukelbir, and Jon D McAuliffe. Variational inference: A review for statisticians. *Journal of the American Statistical Association*, 112(518):859–877, 2017.

Huwenbo Shi, Gleb Kichaev, and Bogdan Pasaniuc. Contrasting the genetic architecture of 30 complex traits from summary association data. *The American Journal of Human Genetics*, 99 (1):139–153, 2016.

Michalis Titsias and Miguel Lazaro-Gredilla. Spike and slab variational inference for multi-task and multiple kernel learning. *Advances in Neural Information Processing Systems*, 24:2339–2347, 2011.

Gao Wang, Abhishek Sarkar, Peter Carbonetto, and Matthew Stephens. A simple new approach to variable selection in regression, with application to genetic fine mapping. *Journal of the Royal Statistical Society Series B: Statistical Methodology*, 82(5):1273–1300, 2020.

Wenmin Zhang, Hamed Najafabadi, and Yue Li. Sparsepro: An efficient fine-mapping method integrating summary statistics and functional annotations. *PLOS Genetics*, 19(12):e1011104, 2023.

Yuxin Zou, Peter Carbonetto, Gao Wang, and Matthew Stephens. Fine-mapping from summary data with the "sum of single effects" model. *PLOS Genetics*, 18(7):e1010299, 2022.
