# Supplementary material for: SharePro: an accurate and efficient genetic colocalization method accounting for multiple causal signals
Source: Bioinformatics. 2024 Apr 30;40(5):btae295. doi: 10.1093/bioinformatics/btae295 (PMC11105950; doi:10.1093/bioinformatics/btae295)
Supplement: btae295_Supplementary_Data [file btae295_supplementary_data.zip › FigS1.pdf]

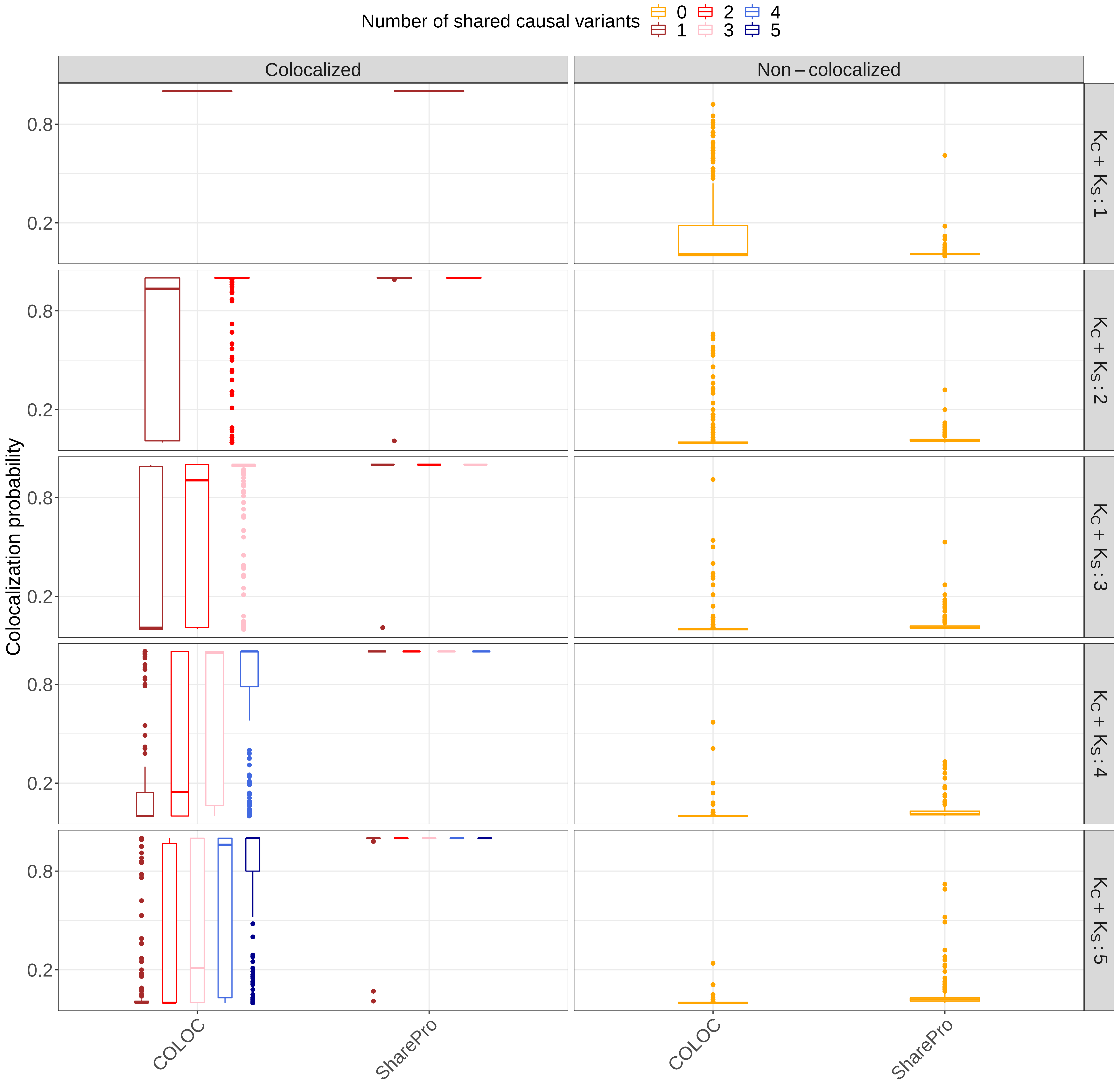

Number of shared causal variants
0
1
2
3
4
5
Colocalized
Non – colocalized
Colocalization probability
0.8
0.2
$K_C + K_S : 1$
$K_C + K_S : 2$
$K_C + K_S : 3$
$K_C + K_S : 4$
$K_C + K_S : 5$
COLOC
SharePro
COLOC
SharePro
