# Supplementary figures and images for: SharePro: an accurate and efficient genetic colocalization method accounting for multiple causal signals
Source: Bioinformatics. 2024 Apr 30;40(5):btae295. doi: 10.1093/bioinformatics/btae295 (PMC11105950; doi:10.1093/bioinformatics/btae295)

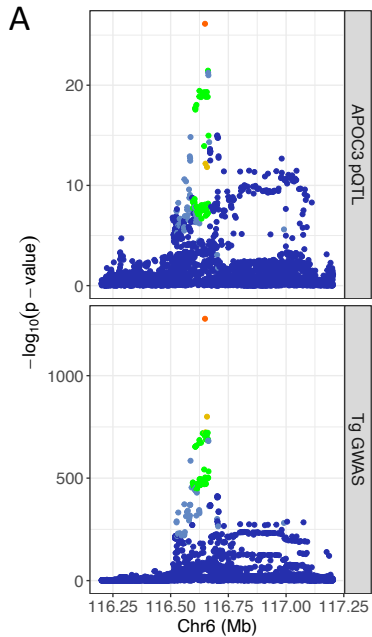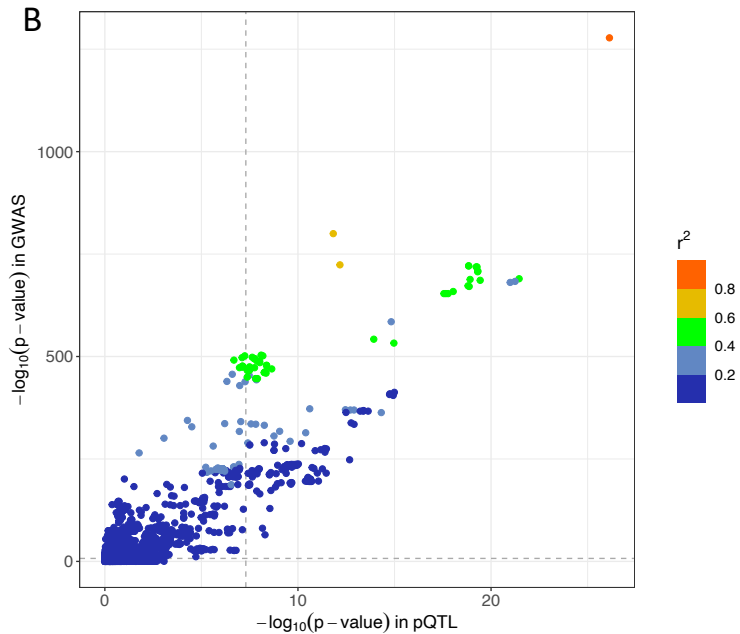

Supplement: btae295_Supplementary_Data [file btae295_supplementary_data.zip › FigS6.pdf]

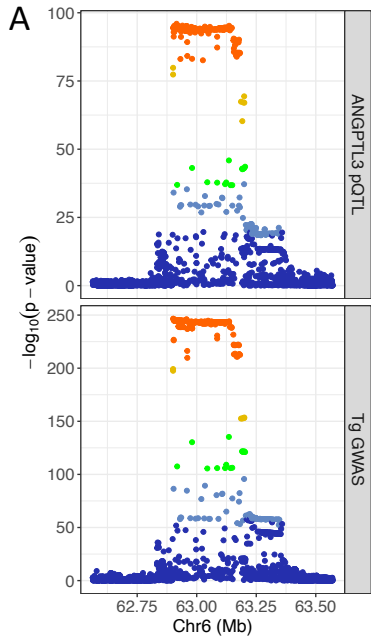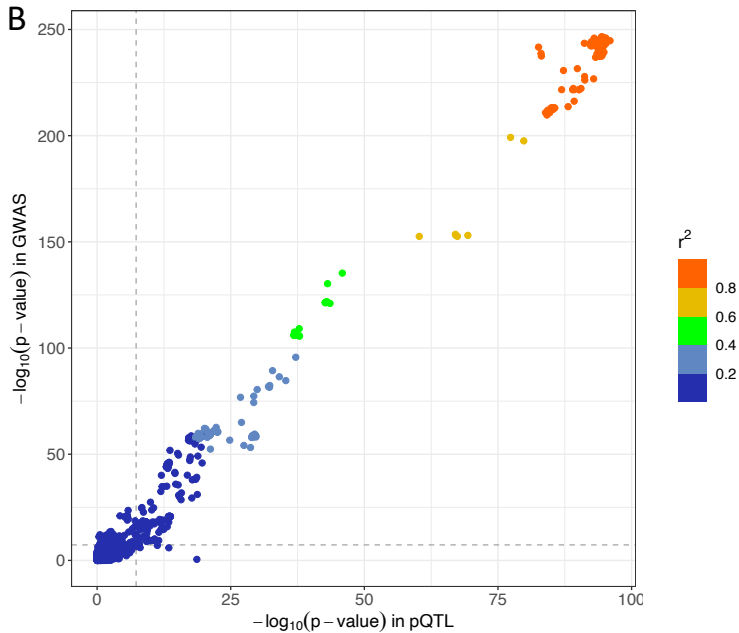

Supplement: btae295_Supplementary_Data [file btae295_supplementary_data.zip › FigS7.pdf]

Number of shared causal variants    1   2   3   4   5

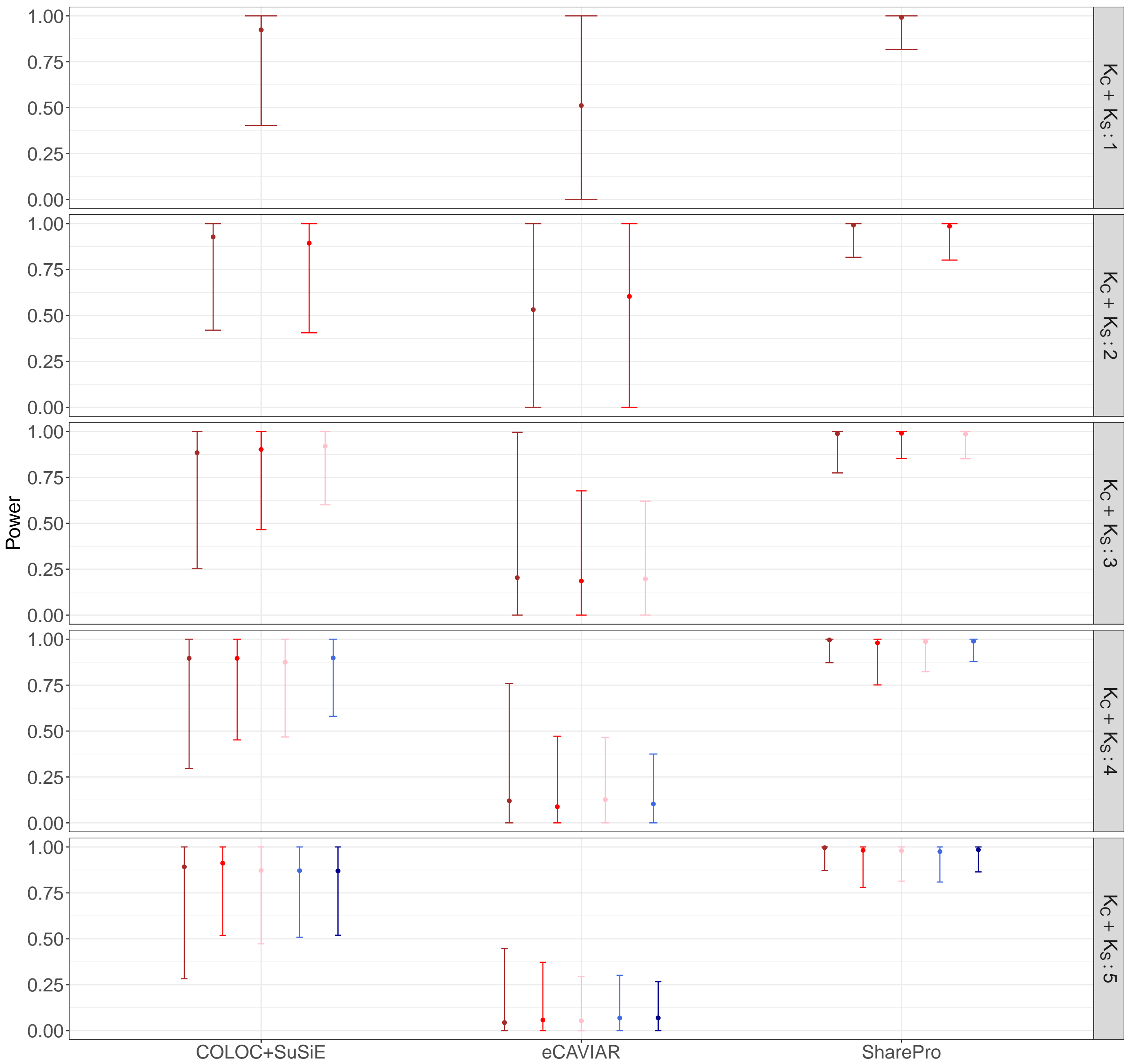

Supplement: btae295_Supplementary_Data [file btae295_supplementary_data.zip › FigS2.pdf]

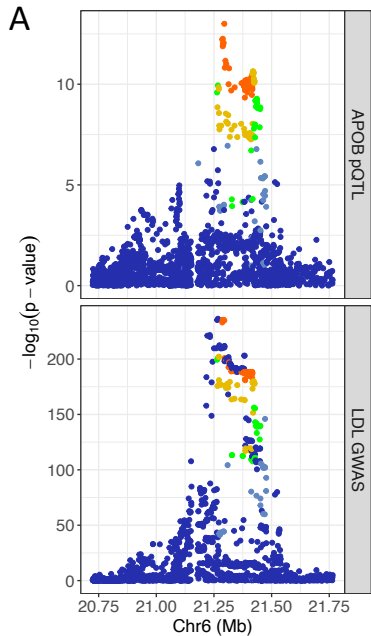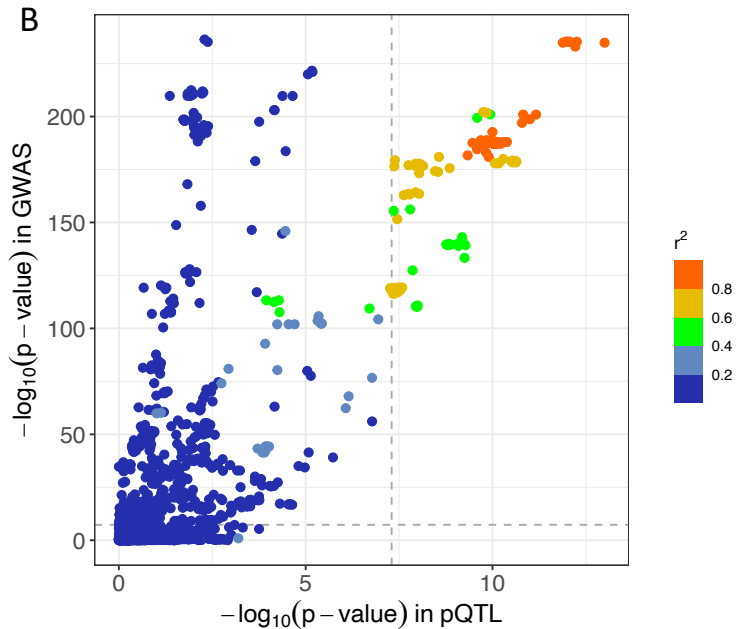

Supplement: btae295_Supplementary_Data [file btae295_supplementary_data.zip › FigS3.pdf]

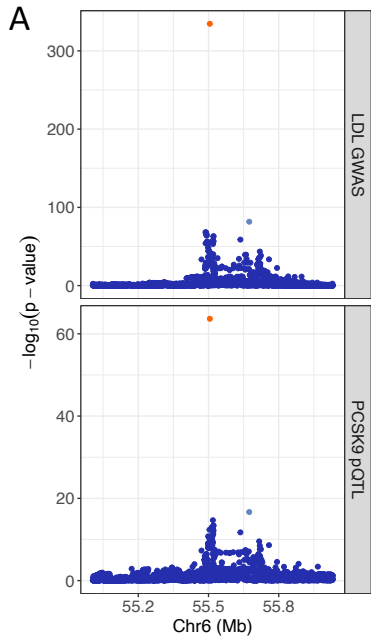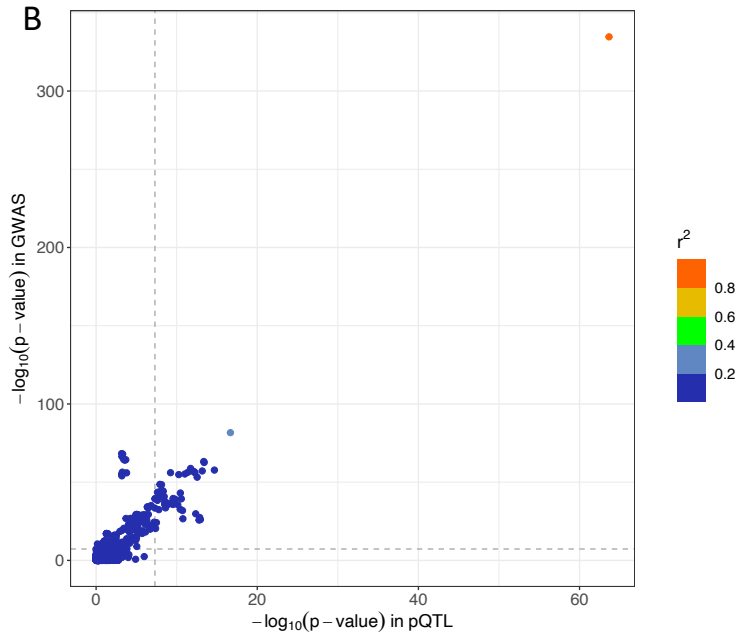

Supplement: btae295_Supplementary_Data [file btae295_supplementary_data.zip › FigS4.pdf]

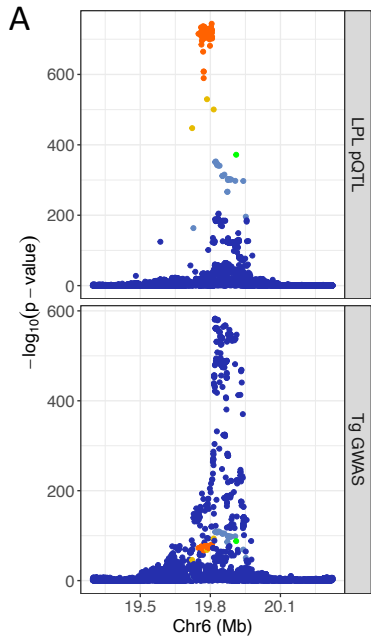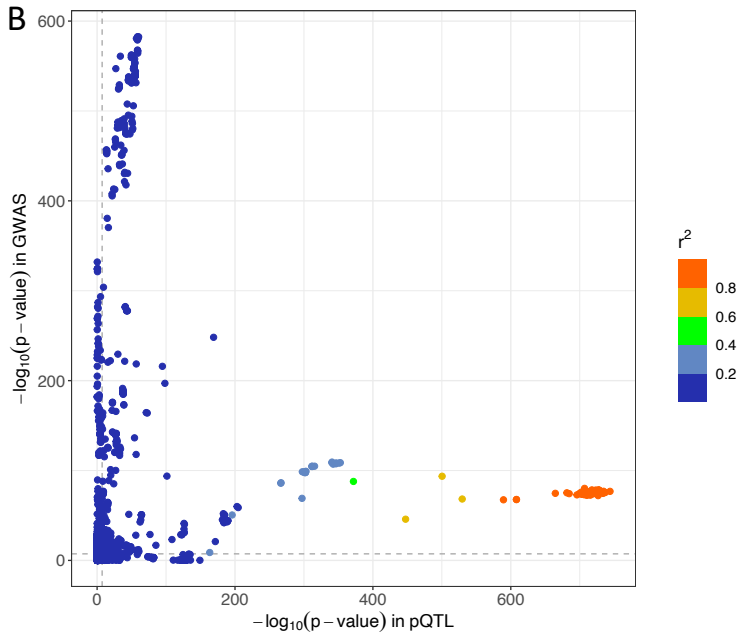

Supplement: btae295_Supplementary_Data [file btae295_supplementary_data.zip › FigS5.pdf]
